# The Current Advances in Design Strategy (Indirect Strategy and Direct Strategy) for Type‐I Photosensitizers

**DOI:** 10.1002/advs.202413365

**Published:** 2024-12-25

**Authors:** Ning Ma, Junjie Wang, Hui Tang, Shiyu Wu, Xiaochun Liu, Kangyao Chen, Yahui Zhang, Xiaoqi Yu

**Affiliations:** ^1^ Department of Chemistry School of Science Xihua University Chengdu 610039 China; ^2^ Sichuan Engineering Research Center for Molecular Targeted Diagnostic & Therapeutic Drugs Chengdu 610039 China; ^3^ Key Laboratory of Green Chemistry and Technology of Ministry of Education College of Chemistry Sichuan University Chengdu 61064 China

**Keywords:** direct strategy, indirect strategy, photodynamic therapy, type‐I photosensitizers

## Abstract

Type‐I photosensitizers (PSs) are among the most potential candidates for photodynamic therapy (PDT), as their low dependence on oxygen endow them with many advantages for treating hypoxic tumor. However, most of the reported type‐I PSs have a contingency of molecular design, because electron transfer (ET) reaction is more difficult to achieve than energy transfer (EET) process. Therefore, it is urgent to understand molecular design mechanisms for type‐I PSs. In this review, the two ways to achieve the type‐I PSs, i.e., inhibiting EET process (type‐II) or enhancing ET process (type‐I), are detailly explained. In response, the current design strategies of type‐I PSs are summarized from two perspectives: indirect strategy (inhibiting EET process: reducing the energy of the lowest triplet excited state (T_1_) to lower than the energy required for the excitation energy transfer to produce singlet oxygen) and direct strategy (enhancing ET process: promoting the ET efficiency of PSs to generate superoxide radicals). The construction of direct strategy can be realized by forming an electron‐rich microenvironment, providing an electron‐deficient intermediate transmitter, and introducing an enhanced electron transfer capacity primitive.

## Graphical abstract

The current design strategies of type‐I PSs are summarized from two perspectives: indirect strategy (inhibiting EET process: reducing the energy of T_1_ to lower than the energy required for the excitation energy transfer to produce singlet oxygen) and direct strategy (enhancing ET process: promoting the ET efficiency to generate superoxide radicals).

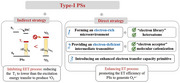

## 1 Introduction

Compared with traditional chemotherapy, radiotherapy, and surgery, photodynamic therapy (PDT) is widely used in many fields such as anti‐tumor and anti‐microbial, due to its minimal invasiveness, low systemic toxicity, negligible drug resistance, and high spatiotemporal selectivity.^[^ ^1^, ^2^, ^3^, ^4^ ^]^ Meanwhile, PDT is also easily combined with other treatments to form multimodal combination therapies, such as photoimmunotherapy,^[^ ^5^, ^6^ ^]^ photothermal therapy (PTT)^[^ ^7^, ^8^ ^]^ and photoacoustic therapy,^[^ ^9^, ^10^ ^]^ which further shows its advanced compatibility.

**Scheme 1:**
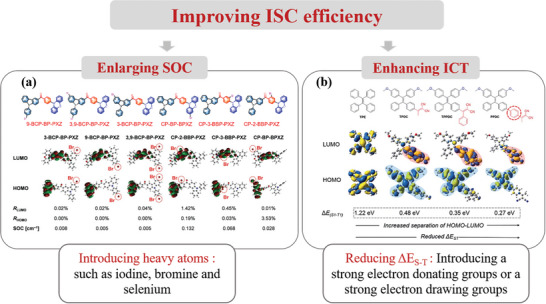
Two ways to improve ISC efficiency. a) Enlarged SOC by introducing heavy atoms. Reproduced with permission.^[^ ^18^ ^]^ Copyright 2022, Wiley‐VCH GmbH. b) Enhanced ICT by reducing ΔE_S‐T_.^[^ ^19^ ^]^ Copyright 2015, Royal Society of Chemistry. Open Access.

The key process involved in PDT is the interaction between photosensitizers (PSs) and molecular oxygen (O_2_) to generate cytotoxic reactive oxygen species (ROS) under light irradiation.^[^ ^11^, ^12^, ^13^, ^14^ ^]^ Upon absorbing photons, PSs were stimulated from the ground state to the excited singlet state and then to a long‐lived triple‐excited state via intersystem crossing (ISC). Therefore, both experimental results and theoretical analysis confirm that efficient ISC is essential for achieving  high ROS yields. In general, there are two main ways to improve ISC processes. The first approach involves enlarging the spin‐orbit coupling (SOC) constant between singlet and triplet states by introducing heavy atoms such as iodine, bromine, and selenium.^[^ ^15^, ^16^, ^17^, ^18^ ^]^ For example, as shown in **Scheme** **1**a, bromine atoms are introduced at different positions in a fluorescent luminescent molecule consisting of a benzoyl receptor and two electron‐donors of phenacazole and phenoxoxazine (PXZ). The theoretical calculation reveals that CP‐2‐BBP‐PXZ, CP‐3‐BBP‐PXZ, and CP‐BP‐BPXZ exhibit larger SOC values, reaching 0.132, 0.068, and 0.028 cm^−1^, respectively, indicating a more efficient SOC interaction between bromine atoms and conjugated molecular skeleton. It is shown that these luminescent elements have effective internal heavy atom effect.^[^ ^18^ ^]^ However, heavy atoms often induce dark toxicity, making them unsuitable for PDT applications. Therefore, the second approach, enhancing the intramolecular charge‐transfer (ICT) state, is developed and studied. The energy level difference between singlet and triplet states (ΔE_S‐T_) can be effectively reduced by augmenting the ICT state. In general, the introduction of strong electron donating groups or strong electron withdrawing groups can achieve strong ICT effects.^[^ ^19^, ^20^, ^21^ ^]^ For example, based on the parent tetraphenylethylene (TPE), by incorporating dicyanovinyl as the electron acceptor and methoxy as the electron donor, TPDC, TPPDC, and PPDC were synthesized, as shown in the Scheme 1b. According to theoretical calculation, the ΔE_S‐T_ values of TPDC, TPPDC, and PPDC are 0.48, 0.35, and 0.27 eV respectively, much smaller than the 1.22 eV of TPE. ROS generation measurement indicate that PPDC has a higher ROS yield, confirming that reducing ΔE_S‐T_ value can improve the ISC efficiency, increase ROS generation, and improve PDT efficacy.^[^ ^19^ ^]^

**Scheme 2:**
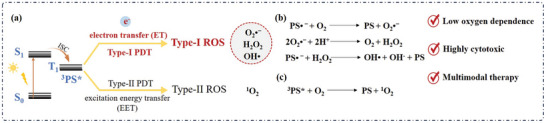
a) Process of type‐I and II PDT. Photophysical and photochemical mechanisms of b) type‐I and c) type‐II processes for ROS generation.

These ROSs can not only directly lead to apoptosis or necrosis of tumor cells, but also rapidly interact with biological substances to induce immunogenic cell death of tumor cells through activating immune cells and triggering immune responses within the tumor microenvironment.^[^ ^22^, ^23^, ^24^, ^25^, ^26^ ^]^ In general, the process of generating ROS can be categorized into two types based on the mechanisms, i.e., type‐I and type‐II (**Scheme** **2**a).^[^ ^26^, ^27^, ^28^, ^29^, ^30^, ^31^ ^]^ Type‐I PSs reach their triplet state (^3^PS^*^) upon excited by lights, followed by an electron transfer (ET) reaction between the adjacent substrate and O_2_ to generate ROS. This reaction leads to the production of superoxide radicals (O_2_·^−^). O_2_·^−^ then interacts with protons (H^+^) to form a second ROS, hydrogen peroxide (H_2_O_2_) and O_2_. In this process, O_2_ is recycled, making type‐I PSs less dependent on the availability of O_2_. Finally, the hydroxyl radicals (OH·) are formed (Scheme 2b).^[^ ^29^, ^30^, ^32^, ^33^, ^34^ ^]^ In contrast, type‐II PSs change the O_2_ to the excited state (^1^O_2_), through an excited energy transfer (EET) between ^3^PS^*^ and the surrounding O_2_ (Scheme 2c).

Compared with type‐I process, type‐II process is more mechanistically favorable and oxygen‐dependent (vide infra). However, the aggressive growth of tumors often leads to a hypoxic microenvironment where O_2_ is rapidly consumed by the metabolic demands of proliferating cells.^[^ ^35^, ^36^, ^37^, ^38^ ^]^ This hypoxic environment severely limits the expected efficacy of type‐II PSs and reduces the clinical efficacy of PDT, due to the highly oxygen‐dependent nature of type‐II process. Over the past decade, improving the ^1^O_2_ concentration in the tumor is the core strategy to offset the stumbling block of type‐II PSs.^[^ ^39^, ^40^, ^41^, ^42^, ^43^ ^]^ While these methods have eased the problem of hypoxia to some extent, they also encountered problems of safety concerns, accelerated cancer cell growth and metastasis, and unpredictable outcomes, challenging the practical application of PDT. To address the issue of hypoxic environment, it is necessary to design and synthesize a new type PSs, which are oxygen insensitive.^[^ ^44^, ^45^, ^46^, ^47^ ^]^ Compared to type‐II, type‐I PDT has the following three advantages: i) reduced oxygen dependence, as type‐I PDT is less dependent on O_2_ due to the oxygen cycle involved the ET process as shown in Scheme 2b.^[^ ^12^, ^48^, ^49^ ^]^ ii) higher cytotoxicity, as the O_2_·^−^ produced by the type‐I process not only destroys tumor cells, but also participates in a catalytic cascade that triggered by superoxide dismutase to produce highly cytotoxic OH·.^[^ ^50^ ^]^ iii) dual‐mode, as OH· can transform protumoral M2 macrophages into antitumoral M1 macrophage, which showed synergistic immunotherapy.^[^ ^51^, ^52^, ^53^ ^]^

**Scheme 3:**
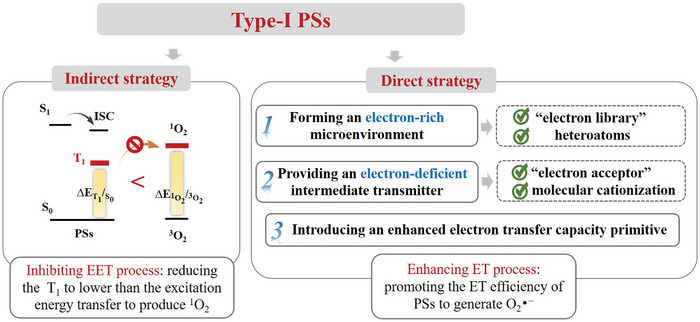
Two strategies: indirect strategy and direct strategy of type‐I PSs.

Strategies for constructing PSs with outstanding properties have been summarized in several excellent review articles and are not discussed here.^[^ ^32^, ^54^, ^55^ ^]^ Unfortunately, type‐I PSs are rarely reported at present, and the design of type‐I PSs remains challenging due to the lack of reliable theoretical guidance. The main reason is that the ET process between ^3^PS^*^ and the substrate can hardly compete with the EET process between ^3^PS^*^ and O_2_. Therefore, either inhibiting EET process or enhancing ET process can enhance the type‐I PDT. Based on that, this review summarized the research progress in the design strategy of type‐I PSs from two perspectives as shown in **Scheme** **3**: indirect strategy (inhibiting EET process: reducing the energy of the lowest triplet excited state (T_1_) below the energy required for EET to produce ^1^O_2_) and direct strategy (enhancing ET process: promoting the ET efficiency of PSs to generate O_2_·^−^). Abbreviations and full name in this paper are listed in **Table** **1**.

**Table 1:** Summary of abbreviations and full name in this paper.

Abbreviation	Full name
PSs	Photosensitizers
PDT	Photodynamic therapy
ET	Electron transfer
EET	Excitation energy transfer
PTT	Photothermal therapy
O_2_	Molecular oxygen
ROS	Reactive oxygen species
ISC	Intersystem crossing
SOC	spin‐orbit coupling
ICT	intramolecular charge transfer
∆E_S‐T_	The lowest singlet−triplet energy gap
TPE	tetraphenylethylene
H_2_O_2_	Hydrogen peroxide
OH·	Hydroxyl radicals
^3^PS*	The excited triplet state of the PS
^1^O_2_	Singlet oxygen
T_1_	The lowest triplet excited state
HOMO	The highest occupied molecular orbital
LUMO	The lowest unoccupied molecular orbital
∆E_S1‐T1_	The energy gap of the lowest singlet‐triplet state
DHR123	dihydrorhodamine 123
HPF	hydroxyphenyl fluorescein
DCFH	2′,7'‐dichlorodihydrofluorescein diacetate
EPR	electron paramagnetic resonance
DMPO	5,5‐dimethyl‐1‐pyrroline‐N‐oxide
S_1_	lowest singlet excited state
PDI	perylenediimides
*O*_H‐L_	The absolute orbital overlap
DHE	dihydroethidium
ESR	electron spin resonance
BSA	bovine serum albumin
BMPO	5‐tert‐butoxycarbonyl 5‐methyl‐1‐pyrroline N‐oxide
k_ISC_	Efficiency of intersystem crossing
CV	cyclic voltammetry
DFT	density functional theory

## 2 Indirect Strategy: Reduced T_1_ State Energy

The indirect strategy is introduced to design and synthesize type‐I PSs by adjusting T_1_ energy of PSs below the energy required for EET to produce ^1^O_2_, thus inhibiting the type‐II process. According to theoretical calculation, the energy gap of ^3^O_2_/^1^O_2_ is 0.98 eV at the CAM‐B3LYP/6‐31G (d, p) level,^[^ ^33^, ^56^, ^57^ ^]^ 1.12 eV at the CASPT2/PCM level^[^ ^49^ ^]^ or 1.75 ev at the TD‐CAM‐B3LYP/def2SVP.^[^ ^58^ ^]^ Therefore, effectively lowering the T_1_ energy below these values is crucial for implementing the indirect strategy. A large range of overlap between the highest occupied molecular orbital (HOMO) and the lowest unoccupied molecular orbital (LUMO) can maximize electron exchange energy, thus enlarges the energy gap of the lowest singlet‐triplet state (∆E_S1‐T1_), and finally the energy of the T_1_ state falls below the energy gap of ^3^O_2_/^1^O_2_.^[^ ^59^, ^60^, ^61^ ^]^

**Figure 1:**
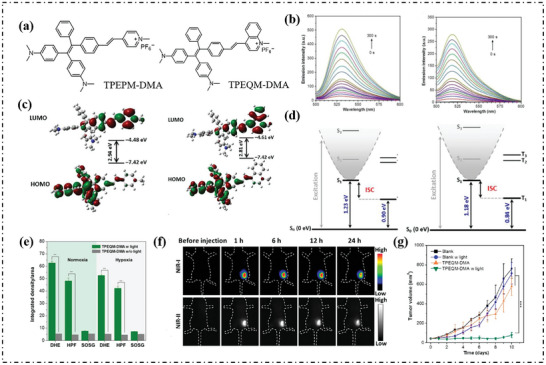
a) Chemical structures of TPEPM‐DMA and TPEQM‐DMA. b) Emission spectra changes of DHR123 (left) or HPF (right) in the presence of 10 µm TPEQM‐DMA in DMSO/PBS mixtures with *f*_PBS_ of 99% after different durations under white light (25 mW cm^−2^). c) Frontier orbital distribution of HOMO and LUMO energy levels of TPEPM‐DMA (left) and TPEQM‐DMA (right) calculated by the CAM‐B3LYP/6‐31G (d, p) basis set. d) The pathway of excited state relaxation and the corresponding energy profile of S_1_ and T_1_ for TPEPM‐DMA (left) and TPEQM‐DMA (right). e) Intracellular ROS detection in CT26 cells using different commercial ROS indicators in the presence of TPEQM‐DMA under normoxia and hypoxia. The corresponding fluorescent intensities extracted from images. The data are presented as means ± standard deviation (*n* = 3), ^***^ *p* < 0.001. Scale bar: 10 µm. f) In vivo time‐dependent NIR‐I and NIR‐II fluorescence images of CT26 tumor‐bearing mice after treating with TPEQM‐DMA. g) The profiles of tumor volume changes of CT26 tumor‐bearing mice during the whole period of TPEQM‐DMA‐mediated type‐I phototheranostics. The data are presented as means ± standard deviation (*n* = 3), ^***^ *p* < 0.001. Reproduced with permission.^[^ ^62^ ^]^ Copyright 2023, American Chemical Society.

Tang's group synthesized two push‐pull cationic PSs used TPE as core, named TPEPM‐DMA and TPEQM‐DMA as shown in **Figure** **1**a.^[^ ^62^ ^]^ The fluorescence intensity of type‐I ROS indicator, dihydrorhodamine 123 (DHR123), and hydroxyphenyl fluorescein (HPF), increased sharply under white light irradiation (Figure 1b), indicating that O_2_·^−^ and OH· were produced. According to the frontier molecular orbitals shown in Figure 1c, the HOMO−LUMO band gap narrates were 2.94 eV (TPEPM‐DMA) and 2.81 eV (TPEQM‐DMA), respectively. It was worth noting that, vertical emission energy from T_1_ to S_0_ (0.90 eV of TPEPM‐DMA and 0.84 eV of TPEQM‐DMA) was less than the oxygen sensitization threshold (0.98 eV), so the EET process was limited (Figure 1d). Then, after treatment of CT26 cells co‐incubation with TPEQM‐DMA, significant fluorescence of indicator enhancement was detected under both normoxic and hypoxic conditions (Figure 1e), which proved that type‐I PSs can effectively overcome the hypoxic barrier. Finally, the in vivo anti‐tumor performance of TPEQM‐DMA particles was evaluated. As shown in Figure 1f, obvious near‐infrared fluorescence signals were observed at the tumor site 1 h after TPEQM‐DMA injection. Eventually, controlled experiments confirmed the tumor inhibition effect of TPEQM‐DMA under white light irradiation (Figure 1g).

**Figure 2:**
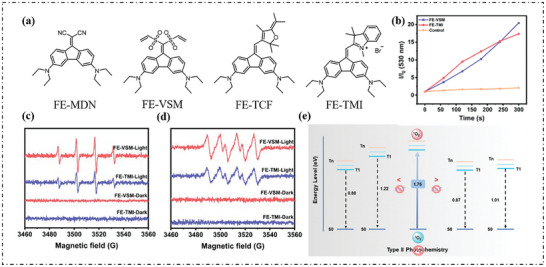
a) Chemical structures of FE‐MDN, FE‐VSM, FE‐TCF, and FE‐TMI. b) Plots of the relative PL intensity of DHR123 in the presence of 10 mm PSs in PBS solution upon green light irradiation for 5 min. c,d) EPR signals of DMPO (for type‐I ROS detection) in the presence of FE‐TMI and FE‐VSM in PBS. e) Energy profile for a putative type‐II energy transfer from the T_1_ levels of FE‐VSM, FE‐MDN, FE‐TCF, and FE‐TMI to ^3^O_2_(TD‐PBE‐D3(BJ)/def2‐TZVP basis set). Reproduced with permission.^[^ ^63^ ^]^ Copyright 2024, Royal Society of Chemistry.

Four type‐I PSs were synthesized by Li's group (**Figure** **2**a).^[^ ^63^ ^]^ DHR123 was employed as the O_2_·^−^ indicator. After adding PSs, the emission intensity was significantly enhanced, and FE‐VSM and FE‐TMI showed outstanding O_2_·^−^ generation efficiency (Figure 2b). To further explore the generation of type‐I ROS, electron paramagnetic resonance (EPR) measurements was performed using 5, 5‐dimethyl‐1‐pyrrolin‐n‐oxide (DMPO) as a spin trap. Distinct and typical EPR signal of O_2_·^−^ and OH· were observed from the irradiated solution of FE‐VSM/FE‐TMI in the presence of DMPO (Figure 2c,d). Theoretical calculations were performed, revealing the vertical emission energy of FE‐VSM (0.88 eV), FE‐MDN (1.22 eV), FE‐TCF (0.87 eV) and FE‐TMI (1.01 eV) from T_1_ to S_0_ were all lower than the oxygen sensitization threshold (1.75 eV). The EET process is thus restricted (Figure 2e).

**Figure 3:**
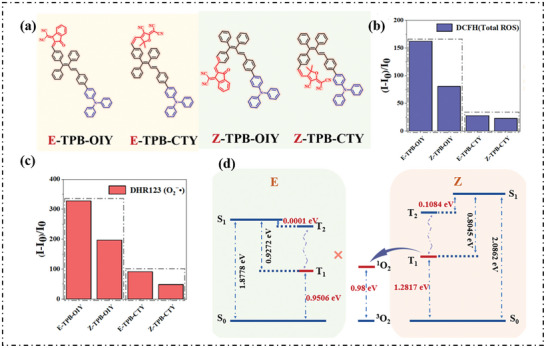
a) Molecule structures of E/Z‐TPB‐OIY and E/Z‐TPB‐CTY. b) PL intensity at 532 nm for the DCFH indicator and c) at 530 nm for the DHR123 indicator with E/Z‐TPB‐OIY and E/Z‐TPB‐CTY upon white light irradiation (2 mW cm^−2^). d) Relative energy levels of E/Z ‐TPB‐OIY calculated. Reproduced with permission.^[^ ^64^ ^]^ Copyright 2024, The Authors. Royal Society of Chemistry.

Our group prepared two pairs of E/Z isomers of tetraphenyl‐1,3‐butadiene (TPB) derivatives (**Figure** **3**a).^[^ ^64^ ^]^ The E isomer demonstrated superior type‐I ROS generation efficiency compared with the Z isomer by inhibiting the EET process. The fluorescence intensity of DCFH showed the order of (Yield of ROS) _E isomer_ > (Yield of ROS) _Z isomer_ (Figure 3b). Similarly, the proportion of type‐I ROS was higher for the E isomer than the Z isomer (Figure 3c). Theoretical calculation showed that the T_1_ energy of E isomer (0.9506 eV) is lower than the oxygen sensitization threshold (0.98 eV), indicating that the EET process between E‐isomer and O_2_ is limited. In contrast, the Z isomer with a T_1_ energy of 1.2817 eV favors the production of ^1^O_2_, thus explaining the higher production of type‐I ROS in the E isomer than the Z isomer (Figure 3d).

A larger ∆E_S1‐T1_ can reduce T_1_ energy, but it will also hinder effective ISC, thus diminishing the PDT effect. It has been reported that an energy gap of less than 0.4 eV (ΔE_S1‐Tn_) is favorable for facilitating efficient ISC processes.^[^ ^65^, ^66^, ^67^ ^]^ While reducing the T_1_ level, the intermediate state is adjusted to complete the effective ISC process. Namely, PSs in the excited state changes from the S_1_ to the T_n_ by ISC, and then relaxes to T_1_ through a rapid diabatic process. Since the S_1_‐T_n_ energy gap is small enough for ISC, and T_1_‐S_0_ energy gap is less than the energy required to generate ^1^O_2_, the generation of ^1^O_2_ generation is prevented.

**Figure 4:**
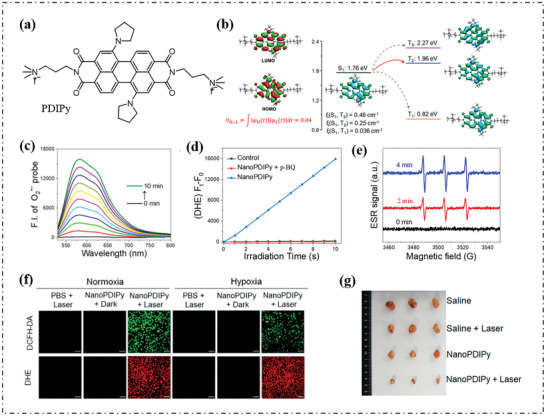
a) Chemical structure of PDIPy. b) Calculated frontier molecular orbitals, energy levels, and the analysis for the distributions of the hole (cyan) and electron (green) of PDIPy in different states. *O*_H‑L_ represents the absolute overlap between the HOMO and LUMO orbitals. c) Time‐dependent fluorescence spectra of DHE (50 µm) in aqueous solution under 730 nm laser irradiation at 0.3 W cm^−2^ in the presence of NanoPDIPy and ctDNA. d) Plots of ΔFl. (*F*_t_ – *F*_0_) of DHE at 580 nm in the presence of NanoPDIPy and NanoPDIPy with p‐BQ in different irradiation times. Water was used as a control. e) ESR signals of 1‐hydroxy‐3‐carboxy‐2,2,5,5‐tetramethylpyrrolidine induced by NanoPDIPy indifferent irradiation times. f) ROS and O_2_·^−^ generation in A549 cells mediated by NanoPDIPy in normoxia and hypoxia with DCFH‐DA and DHE as the probes, respectively. Scale bars = 50 µm. g) Photographs of the excised tumors at the end of therapy. Reproduced with permission.^[^ ^68^ ^]^ Copyright 2023, American Chemical Society.

Following this strategy, Gou's group designed and synthesized type‐I PSs named PDIPy by enlarge ∆E_S1‐T1_ to reduce T_1_ while still maintaining effective ISC from S_1_ to T_2_.^[^ ^68^ ^]^ The structure was illustrated in **Figure** **4**a. Since pyrrolidines have minimal effect on electron configuration, the molecular orbitals of HOMO and LUMO were mainly located on the perylenediimides (PDI) core (Figure 4b), indicating a large spatial overlap between HOMO and LUMO. The absolute orbital overlap (*O*_H‐L_) was determined to be 0.84, producing a large electron exchange energy and ∆E_S1‐T1_. In that case, the energy of the T_1_ (0.82 eV) was lower than the oxygen sensitization threshold of 0.98 eV. The energy gap between S_1_ and T_2_ was significantly less than 0.4 eV, and the spin‐orbit coupling constant (ξ) between S_1_ and T_2_ was 0.25 cm^−1^, thus an efficient ISC process can occur from S_1_ to T_2_. PDIPy were used to prepare nanoparticles (NanoPDIPy), whose O_2_·^−^ generation ability was further evaluated by dihydroethidium (DHE) and electron spin resonance (ESR) spectroscopy (Figure 4c–e). Experimental results confirmed that the NanoPDIPy can still produce a large amount of ROS in cell under low oxygen conditions with laser irradiation, as shown in Figure 4f. Finally, the anti‐cancer effects of NanoPDIPy‐mediated PDT and PTT on A549 tumor‐bearing mice were detected. NanoPDIPy+Laser treatment showed strong antitumor activity compared to other groups (Figure 4g).

**Figure 5:**
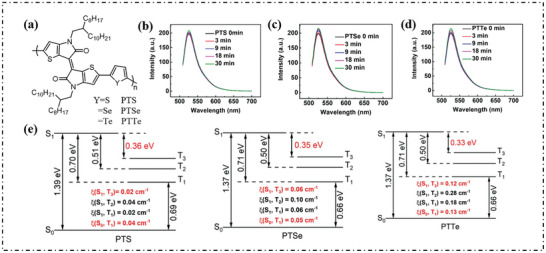
a) Chemical structures of PTS, PTSe, and PTTe. Fluorescence spectra changes of DHR123 induced by PTS b), PTSe c), and PTTe d) NPs. e) Excitation energies of the low‐lying excited states and spin‐orbit coupling constants (ξ (Sn, Tn)) between them based on the optimized S_0_‐geometries of PTS, PTSe, and PTTe oligomers (repeat unit is 3) calculated by TDA‐B3LYP. under the 1064 nm laser irradiation. Reproduced with permission.^[^ ^57^ ^]^ Copyright 2022, Wiley‐VCH GmbH.

Huang's group used the donor‐acceptor (D‐A) strategy to construct three narrow‐band gap semiconductor polymers (PTS, PTSe, and PTTe), using thiophene as the strong electron‐acceptor unit, and thiophene (S), selenophene (Se) or tellurophene (Te) as the electron donor unit (**Figure** **5**a).^[^ ^57^ ^]^ The authors calculate the energy levels of HOMO/LUMO (E_HOMO_/E_LUMO_). The E_HOMO_/E_LUMO_ values were −5.217/−4.291 eV for PTS, −5.190/−4.289 eV for PTSe, and −5.135/−4.254 eV for PTTe, respectively. Obviously, tuning chalcogenide copolymers narrowed the band gap between the HOMO and LUMO and improved the efficiency of intramolecular charge transfer. The fluorescence of DHR123 increased progressively (Figure 5b–d), indicating that all three polymers had the ability to produce O_2_·^−^. The ∆E_S‐T_ between S_1_ and the nearest T_3_ was gradually reduced, from 0.36 eV for PTS, 0.35 eV for PTSe, and 0.33 eV for PTTe, respectively, resulting in effective ISC. It should be noted that the T_1_ (0.69, 0.66, and 0.66 eV) of PTS, PTSe, and PTTe nanoparticles (NPs) were below the 0.98 eV threshold for oxygen sensitization, which severely limits the type‐II PDT process (Figure 5e).

**Figure 6:**
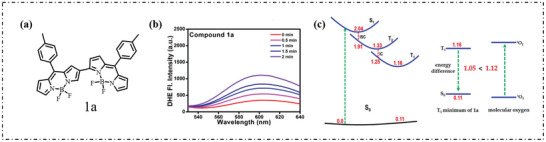
a) Chemical structure of α‐β‐linked BODIPYs compounds 1a. b) Photoinduced O_2_·^−^ generation ability of compound 1a (10 µm) determined with DHE (30 µm) as a fluorescence probe in DMSO containing 200 µm ctDNA. All fluorescence spectra were determined after photoirradiation with white LED light source (20 mW cm^−2^, excited at 500 nm). c) Excited‐state relaxation pathway from the initially populated S_1_ to T_1_ states of compound 1a mediated by an intermediate T_2_ state. Energy profile for the type‐II energy transfer from the T_1_ species of 1a to ^3^O_2_. Also shown are relevant energies (in eV) calculated at the CASPT2/PCM level. Reproduced with permission.^[^ ^49^ ^]^ Copyright 2021, Wiley‐VCH GmbH.

Yang's group synthesized an α, β‐linked boron dipyrromethene BODIPY dimer (structure shown in **Figure** **6**a).^[^ ^49^ ^]^ As shown in Figure 6b, the fluorescence of DHR123 was significantly enhanced, indicating the formation of O_2_·^−^. Figure 6c shows the effective relaxation pathway of excited states from the initially filled S_1_ to T_1_. It can be seen that compound 1a would quickly relax to the minimum value of S_1_ and further decay to the intermediate T_2_ through the ISC process. This process worked because there was a very small S_1_‐T_2_ energy gap of 0.13 eV at the S_1_ minimum. In T_2_, the system will first relax to its minimum energy level and then continue to decay to the T_1_ state, followed by a T_2_‐T_1_ internal transition process near the T_2_ minimum T_2_‐T_1_ energy gap estimated at 0.08 eV (at the CASPT2/PCM level). The minimum emission energy of the vertical T_1_‐S_0_ was 1.05 eV, which is slightly less than the energy required to excite ^3^O_2_ to ^1^O_2_ (1.12 eV).

The indirect strategy, which inhibits EET process by reducing the energy of T_1_ to lower than the energy required for EET to produce ^1^O_2_, enables PSs to generate type‐I ROS effectively. However, it has a certain limitation due to the unpredictable nature of triple energy levels. Although some reliable strategies have been developed for regulating T_1_ energy, there is a lack of clear structural strategies for higher T_n_ energies. Therefore, direct strategies, enhanced ET process are increasingly studied.

## 3 Direct Strategy: Enhance Electron Transfer Process

The core of the type‐I PDT mechanism is the ET process. There are two main pathways to generate O_2_·^−^. i) ^3^PS^*^ abstracts an electron from the surrounding environment, and then transfers that electron to O_2_ to form O_2_·^−^. Therefore, the construction of an electron‐rich environment is conducive to the occurrence of the above ET process. ii) ^3^PS^*^ transfers electrons to an electron‐deficient substance in the environment, which then transfer electrons to O_2_. In this pathway, the electron‐deficient substance act as an intermediary for transferring electron from PSs to O_2_. Based on the above two approaches and regarding electron transfer ability as the key point, the construction of type‐I PSs can be realized by forming an electron‐rich microenvironment, providing an electron‐deficient intermediate transmitter. Also, introducing an enhanced electron transfer capacity primitive is the most direct and effective strategy, among which quinone and its derivatives are the most potential candidates.

**Scheme 4:**
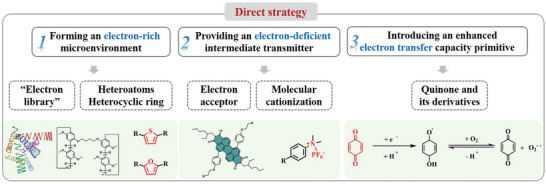
Three key construction methods for type‐I PSs indirect strategy.

Three key construction methods were concluded in **Scheme** **4**.

- Forming an electron‐rich microenvironment using bovine serum albumin (BSA), bispillar [5] arene (BP5A), heteroatoms and heterocyclic rings;
- Providing an electron‐deficient intermediate transmitter using electron acceptors and molecular cationization;
- Introducing an enhanced electron transfer capacity primitive through quinone and its derivatives.

### 3.1 Forming Electron‐Rich Microenvironment

The electron‐rich environment helps to promote the efficiency of electron transfer between the PSs and surrounding molecules, promoting the generation of ROS, and thus improving the effect and performance of type‐I PDT. An electron‐rich environment can be created by providing an “electron library” and incorporating heteroatoms or heterocycle.

**Figure 7:**
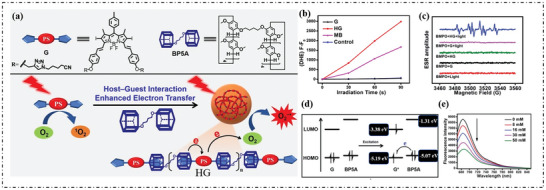
a) The fabrication of HG and photo‐induced generation of ROS. b) Plots of ∆F*_l_*. (F‐F_0_) for DHE at 580 nm upon light irradiation (660 nm, 20 mW cm^−2^) for different time intervals in the presence of G, HG or methylene blue. c) ESR spectra to detect O_2_·^−^generated by G (0.5 mm) and HG (0.5 mm) under illumination, using 5‐tert‐butoxycarbonyl 5‐methyl‐1‐pyrroline N‐oxide (BMPO) (25 mm) as a spin trap. d) Schematic diagram of the electron transfer process between G and BP5A through HOMO and LUMO energy levels. e) The emission spectra of G (10 µm) at 25 °C in acetonitrile with increasing amounts of BP5A (0–50 mm) under excitation at 650 nm.^[^ ^59^ ^]^ Copyright 2022, Wiley‐VCH GmbH. Open Access.

Yang's group realized the transformation of traditional type‐II PS into type‐I PS by introducing the electron‐rich substrate.^[^ ^59^ ^]^ Iodide BODIPY(G), a typical type‐II PS and electron‐rich BP5A were used to prepare supramolecular PSs (HG), as illustrated in **Figure** **7**a. The electron transfer was enhanced because BP5A provide an electron‐rich environment. As shown in Figure 7b, the fluorescence of DHE significantly increased indicating that HG can effectively produce O_2_·^−^. Further, the signal in ESR spectroscopy matched well with the O_2_·^−^ (Figure 7c). The HOMO (LUMO) of G and BP5A were −5.19 eV (−3.38 eV) and −5.07 eV (−1.31 eV), respectively. As shown in Figure 7d, electron‐rich BP5A acted as an electron donor, transferring electrons to the excited state of G to form G^·−^, which subsequently transfer electrons to O_2_ and produced O_2_·^−^. In order to further investigate the occurrence of photoinduced electron transfer, the quenching effect of BP5A on G emission was studied. With the addition of BP5A, the emission of G was gradually quenched (Figure 7e).

**Figure 8:**
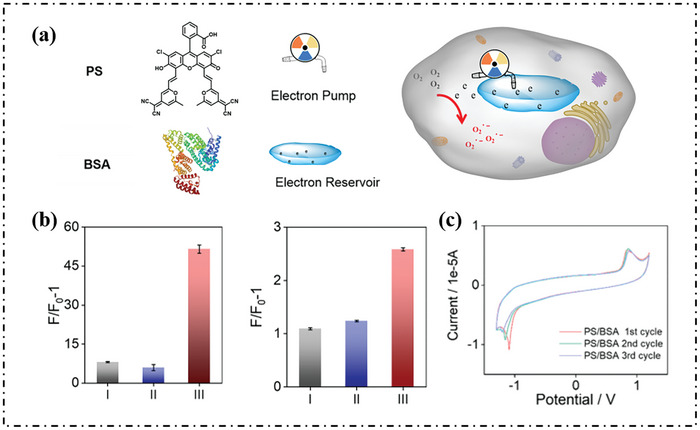
a) Simple schematic diagram of the photosensitizer PS acting as an “electron pump” and BSA as an “electron reservoir” in the type‐I PDT process. b) Plots of relative integration fluorescence intensity of DCFH (left) and DHR123 (right) in the three systems, I: PS (5 µm) in PBS, II: PS (5 µm) in PBS with 30% EtOH, III: PS (5 µm) and BSA (2.5 µm) in PBS. c) Four cycles of cyclic voltammograms of PS after BSA was added in nitrogen‐saturated PBS containing 20% DMF in volume, which were recorded at 0.05 V s^−1^ using a three‐electrode system (all potentials were referenced to NHE via an external ferrocene calibration). Reproduced with permission.^[^ ^69^ ^]^ Copyright 2023, American Chemical Society.

Peng's group proposed a strategy, which successfully converted heat‐activated delayed fluorescence materials to type‐I PSs by constructing an electron‐rich environment using BSA as an “electron reservoir” (**Figure** **8**a).^[^ ^69^ ^]^ The author further confirmed that type‐I ROS (O_2_·^−^) was the predominant ROS generated among PS/BSA (Figure 8b). In order to evaluate the “electron reservoir” ability of BSA, electrochemical experiments were carried out. When BSA was added to the PS solution, the reduction current gradually decreased with the electrochemical scanning cycle number increased (Figure 8c). The current changes indicated that an electrochemically inactive complex may from between PS and BSA.

By incorporating electron rich structures (BP5A and BSA), the electron transfer ability among multiple substances is increased, successfully yielding a type‐I PSs system. However, the assembly of PSs and electron‐rich substrates is crucial in this protocol, which requires careful consideration of their energy level matching, distance, and interaction. Therefore, the strategy of introducing heteroatoms (heterocycle) into molecules through chemical bonding is increasingly studied because the lone pair electrons from heteroatoms can enhance ISC, facilitate electron transfer, and eventually lead to increased type‐I ROS production.

**Figure 9:**
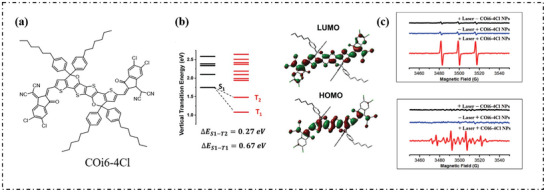
a) Chemical structure and b) the HOMO–LUMO distribution, the energy levels, and ∆E_ST_ values of the COi6‐4Cl molecule. c) EPR spectra of 2,2,6,6‐tetramethyl‐4‐piperidone /^1^O_2_ (up) and DMPO/OH· (down) for COi6‐4Cl NPs (20 µm according to COi6‐4Cl) under 880 nm irradiation at 0.3 W cm^−2^ for 15 min. Reproduced with permission.^[^ ^14^ ^]^ Copyright 2020, Wiley‐VCH GmbH.

Tian's group reported a highly efficient type‐I ROS reagent (COi6‐4Cl) by introducing heteroatoms (structure shown in **Figure** **9**a).^[^ ^14^ ^]^ The electron‐rich O and S atoms significantly improved the electron donating capability. To understand the ET process, the electronic structure of COi6‐4Cl was analyzed by theoretical calculation (Figure 9b). It was found that the LUMO was delocalized along the entire molecular backbone, while the HOMO was localized at the donor core. The strong A‐D‐A structure led to HOMO‐LUMO separation, so that ΔE_S1‐T2_ values were 0.27 eV, improving an efficient intersystem crossing (k_ISC_) and PDT efficiency. As shown in Figure 9c, the EPR spectra showed triplex signals of 1:1:1:1 and 1:2:2:1. For COi6‐4Cl, the electron‐rich oxygen atoms significantly improve the electron‐donating capability of the donor core, which combines with the strong electron‐withdrawing chloride substituted dicyanovinylindanone, resulting in an intense intramolecular charge transfer.

**Figure 10:**
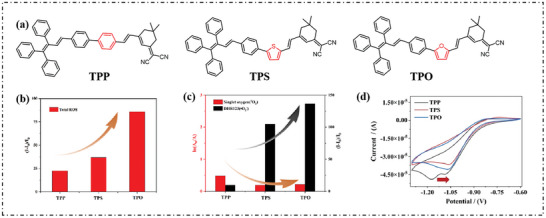
a) Chemical structures of TPP, TPS, and TPO. b) Plots of relative integration fluorescence intensity for the DCFH indicator with TPP, TPS, and TPO upon white light irradiation (2 mW cm^−2^), [DCFH] = 40 µm. c) Plots of relative integration fluorescence intensity for the DHR123 and ABDA indicators with TPP, TPS, and TPO upon white light irradiation (2 mW cm^−2^), [DHR123] = [ABDA] = 40 µm. [TPP] = [TPS] = [TPO] = 10 µm. d) The 90th cyclic voltammogram of TPP, TPS, and TPO in DCM with 0.1 m (n‐Bu)_4_N^+^PF_6_^−^ as the supporting electrolyte, Ag/Ag^+^ as the reference electrode, a glassy‐carbon electrode as the working electrode, and a Pt wire as the counter electrode; scan rate, 50 mV s^−1^. Reproduced with permission.^[^ ^70^ ^]^ Copyright 2024, The Authors. Royal Society of Chemistry.

Our group designed and synthesized three PSs (TPP, TPS, and TPO) through heterocyclic strategy, and structures are shown in **Figure** **10**a.^[^ ^70^ ^]^ The presence of electron‐rich heterocyclic rings effectively increased the proportion of type‐I PSs. As shown in Figure 10b, the ROS yield followed the order (Yield of ROS) _TPO_ > (Yield of ROS) _TPS_ > (Yield of ROS) _TPP_, with a similar trend for the proportion of O_2_·^−^ (Proportion of O_2_·^−^) _TPO_ > (Proportion of O_2_·^−^) _TPS_ > (Proportion of O_2_·^−^) _TPP_ (Figure 10c). Electrochemical experiments were carried out by cyclic voltammetry (CV) to prove the ability of heterocyclic rings to increase electron transfer. PSs all showed obvious reduction peaks, and the lower ground state reduction potential *E*_red_ (PS/PS^−^·) was slightly enhanced to be lower than −0.33 V (*E*_red_ (O_2_/ O_2_·^−^) = −0.33 V, Figure 10d). The anode displacement observed in the heterocyclic “bridge” compared to benzene suggests that TPO had a greater tendency to accept electrons. After that, TPO NPs were applied to in vitro and in vivo PDT and had good tumor treatment abilities.

**Figure 11:**
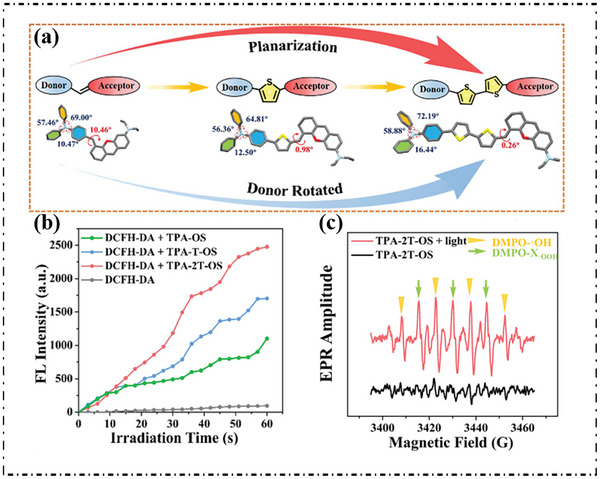
a) Schematic illustration of molecular engineering of TPA‐OS, TPA‐T‐OS, and TPA‐2T‐OS. b) Comparison of the FL intensity at 530 nm of DCFH‐DA alone and DCFH‐DA with these PSs upon NIR‐laser (720 nm, 1 W cm^−2^) irradiation. c) ESR spectra of DMPO exposed to NIR‐laser irradiation of TPA‐2T‐OS. Reproduced with permission.^[^ ^71^ ^]^ Copyright 2022, Royal Society of Chemistry.

Zhou's group prepared a series of D‐π‐A systems (TPA‐OS, TPA‐T‐OS, and TPA‐2T‐OS) with trianiline (TPA) and ammonium salt (OS), respectively (**Figure** **11**a).^[^ ^71^ ^]^ For these PSs (TPA‐T‐OS, TPA‐2T‐OS), thiophenyl groups were introduced simultaneously as π‐bridge to optimize planarization and donor rotation. As shown in Figure 11b, compared with DCFH‐DA, the fluorescence emission of DCFH‐DA increased rapidly in the presence of TPA‐OS, TPA‐T‐OS, or TPA‐2T‐OS, indicating that they could all produce ROS under light irradiation. In addition, the detection of type‐I ROS by 5,5‐dimethyl‐1‐pyrroline N‐oxide (DMPO) under near‐infrared laser irradiation showed that all PSs with DMPO showed free radical signals (Figure 11c), among which TPA‐2T‐OS showed more pronounced OH· signals, proving that the enhanced electron transfer ability in the electron‐rich environment created by heterocyclic ring benefits the proportion of type‐I PSs.

### 3.2 Introduce an Electron‐Deficient Intermediate Transmitter

PSs tend to transfer electrons toward electron‐deficient substances, thereby enhancing electron transfer ability to form O_2_·^−^ and OH·. Therefore, the introduction of electron‐deficient structure intermediates can effectively convert type‐II to type‐I PSs, or increase the proportion of type‐I ROS. The design and synthesis of electron‐deficient substances can be divided into two categories, i.e., electron acceptor strategy and molecular cationization strategy. Electron acceptor strategy introduces electron acceptor as electron‐deficient intermediate transmitter, which shortens the distance between the electron donor and the acceptor, and improves the electron transfer efficiency. PSs were close to an electron acceptor with an appropriate potential and transfer electrons to the electron acceptor and subsequently to O_2_, resulting in efficient production of O_2_·^−^. On the other hand, molecular cationization strategy can not only enhance the electron acceptance capacity of the cationic part and promote ISC, but also increase the electron separation and transfer process.

**Figure 12:**
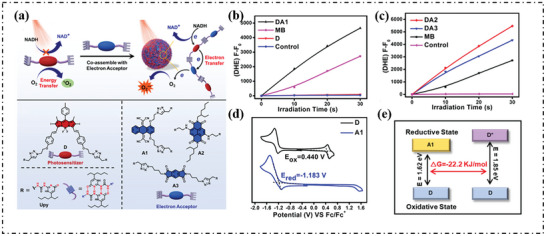
a) Schematic of photodynamic therapy with supramolecular photosensitizers. Plots of ΔF*_l_*. (F‐F_0_) of DHE at 580 nm upon light irradiation (660 nm, 20 mW cm^−2^) for different time intervals in the presence of DA1 b), DA2, DA3 c), or MB. d) D and A1 in DCM with 0.1 m (n‐Bu)_4_N^+^PF_6_^−^ as a supporting electrolyte, Ag/Ag^+^ as a reference electrode, glassy‐carbon electrode as a working electrode, and Pt wire as a counter electrode; scan rate, 100 mV s^−1^; Fc/Fc^+^ was used as an external reference. e) Gibbs free energy of electron transfer between D and A1.^[^ ^72^ ^]^ Copyright 2022, Nature. Open Access.

Yang's group used three different types of electron acceptors (A1, A2, A3), acting as electron‐deficient intermediate transmitter, which can be co‐assembled with conventional type‐II photosensitizers (D) to form supramolecular PSs system (DA1, DA2, or DA3), as shown in **Figure** **12**a.^[^ ^72^ ^]^ The fluorescence of DHE solution significantly increased in the presence of DA1, DA2, or DA3 (Figure 12b,c), indicating that these three systems can effectively produce O_2_·^−^. In order to further study intramolecular electron transfer, the oxidation potential of D and the reduction potential of A1 were determined by cyclic voltammetry (CV). The oxidation potential of D was +0.440 V, and the reduction potential of A1 was −1.183 V (Figure 12d). According to the Rehm–Weller equation, the Gibbs free energy (ΔG) for electron transfer between D and A1 was estimated to be −22.2 kJ mol^−1^ (Figure 12e), confirming that intermolecular electron transfer is thermodynamically feasible.

**Figure 13:**
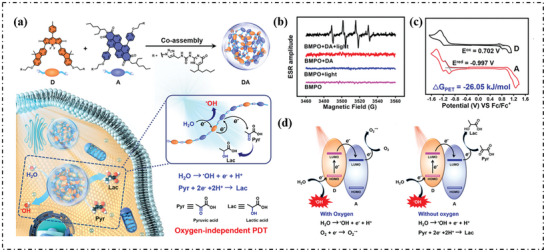
a) Schematic illustration of the preparation of DA, as well as photoinduced generation of OH· and reduction of pyruvic acid. b) ESR spectra to detect OH· generated by DA (0.1 mm) under illumination, using BMPO (25 mm) as a spin trap agent, O_2_ as the electron acceptor. c) Cyclic voltammogram of D and A in DCM with 0.1 m (n‐Bu_)4_N^+^PF_6_^−^ as a supporting electrolyte, Ag/Ag^+^ as a reference electrode, glassy‐carbon electrode as a working electrode, and Pt wire as a counter electrode; scan rate, 100 mV s^−1^; Fc/Fc^+^ was used as an external reference. d)Schematic illustration of the mechanism of the generation of OH· with or without oxygen. Reproduced with permission.^[^ ^1^ ^]^ Copyright 2023, American Chemical Society.

Yang's group further used the type‐II PS, BODIPY (D), and electron‐deficient intermediate transmitter peralkyne diimide (A), to form type‐I supramolecular system (DA, **Figure** **13**a).^[^ ^1^ ^]^ The signal in ESR spectroscopy matched well with the OH· (Figure 13b). To understand the intramolecular electron transfer, CV was employed to determine the oxidation potential of +0.702 V for D and the reduction potential of −0.997 V for A, and calculated the ΔG of photoinduced electron transfer to be −26.05 KJ mol^−1^ (Figure 13c), suggesting that intermolecular electron transfer was thermodynamically feasible. The mechanism of photoinduced OH· production by DA system under normal oxygen and anaerobic conditions was inferred, as shown in Figure 13d. D was excited to the excited state (D^*^) and then electrons were rapidly transferred to the nearby electron‐deficient A to form free radical ion pairs (D^+·^ and A^−·^). Also, D^+·^ can obtain electrons directly from the water molecules, thus oxidizing the water to produce OH·.

**Figure 14:**
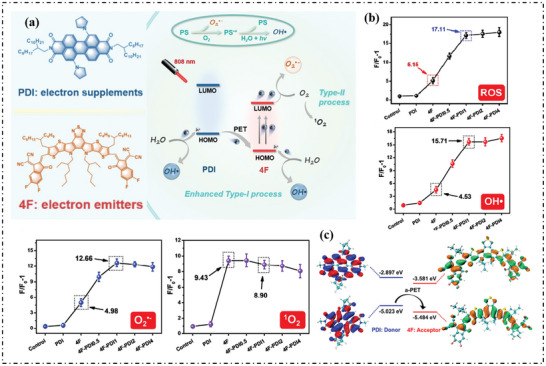
a) Schematic representation of augmented type‐I photosensitization processes by facilitating the separation of electron‐hole pairs. b) The ROS, OH·, O_2_·^−,^ and ^1^O_2_ generating capacities of these NPs were quantitatively compared under 808 nm excitation. c) The HOMO‐LUMO distribution of PDI and 4F and the proposed mechanism of the intermolecular electron transfer process. Reproduced with permission.^[^ ^73^ ^]^ Copyright 2024, Wiley‐VCH GmbH.

Huang's group proposed an acceptor‐triggered photoinduced electron transfer strategy by co‐assembling two organic molecules: PDI and L8‐BO‐EH‐4F (4F).^[^ ^73^ ^]^ This approach harnessed the electron‐deficient properties of PDI to facilitate electron transfer from 4F, resulting in the production of type‐I ROS (**Figure** **14**a). As shown in Figure 14b, 4F can generated both type‐I and type‐II ROS. When PDI was introduced, the obtained co‐assembler (4F‐PDIx NPs) can significantly enhance the ROS production efficiency, and the introduction of PDI can promote the production of OH· and O_2_·^−^ by 3.5‐ and 2.5‐fold, respectively, concluding that the introduction of PDI can promote the production efficiency of type‐I ROS. Theoretical calculation and ultrafast femtosecond transient spectral analysis were used to further elucidate the mechanism. As shown in Figure 14c, the band gap of PDI and 4F was 2.126 and 1.903 eV, respectively. Light irradiation excited the receptor 4F to an excited state, and the resulting active 4F can transfer electrons to O_2_, producing O_2_·^−^. Meanwhile, the electron‐deficient intermediate transmitter PDI easily donated electrons to the excited state of 4F. The resulting cationic free radical immediately captured electrons from surrounding water, oxidizing the molecular water to release OH·.

Consistent with the strategy of creating electron‐rich environment, the additional introduction of another substance as an electron‐deficient intermediate transmitter requires high matching degree of the two molecules. Cationization can provide missing electron gaps and improve electron transport capacity.

**Figure 15:**
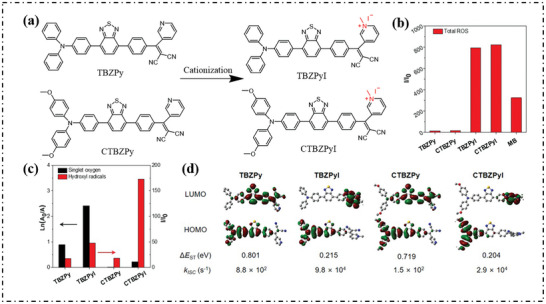
a) Schematic illustration of the cationization molecular engineering strategy for boosting PDT performance. b) DCFH fluorescence enhancement in the presence of AIE PSs in 1 × PBS solution (with 1 vol % DMSO) under light irradiation (20 mW cm^−2^). c) Summary of different ROS generation of these AIE PSs. d) HOMO and LUMO distribution of DTPAPy and DTPAPyPF_6_ calculated by DFT (B3LYP/6‐31G (d, p)). e) Energy levels distribution of excited singlet and triplet states for DTPAPy and DTPAPyPF_6_ calculated by the level of DFT (B3LYP/6‐31G (d, p)). Reproduced with permission.^[^ ^74^ ^]^ Copyright 2022, American Chemical Society.

Tang's group confirmed the feasibility of cationization through the changes of ROS species after the cationization of two molecules (from TBZPy to TBZPyI, and from CTBZPy to CTBZPyI), as shown in **Figure** **15**a.^[^ ^74^ ^]^ It was found that compared with TBZPy and CTBZPy, TBZPyI and CTBZPyI could not only improve the generation efficiency of ROS, but also increase the proportion of OH· (Figure 15b,c). The ΔE_S‐T_ values of TBZPy, TBZPyI, CTBZPy, and CTBZPyI were 0.801, 0.215, 0.719, and 0.204 eV, respectively, as shown in Figure 15d. Cationization could greatly promote the ISC process and promote the generation of T_1_ states, thereby comprehensively improving the generation efficiency of ROS. At the same time, the strong donor‐acceptor system brought by cationization provided an efficient charge transfer process, which facilitated the generation of free radicals through electron transfer.

**Figure 16:**
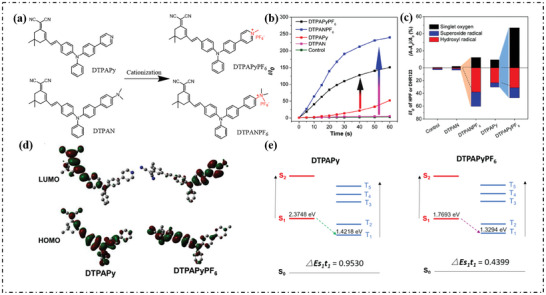
a) Schematic illustration of the cationization molecular engineering strategy. b,c) summary of different types of ROS generation. d) HOMO and LUMO distribution, e) ΔE_S‐T_, and k_ISC_ values of four AIE photosensitizers calculated by TD‐DFT at the basis set level of 6–31 G (d, p). Reproduced with permission.^[^ ^75^ ^]^ Copyright 2022, Elsevier.

Similarly, cationization played a similar role in the DTPAN and DTPAPy molecules (**Figure** **16**a).^[^ ^75^ ^]^ The order of ROS generating capacity that DTPANPF_6_ > DTPAPyPF_6_ > DTPAPy > DTPAN ≈ control showed the DCFH enhancement rate (Figure 16b). The production of ROS in DTPAPyPF_6_ and DTPANPF_6_ clearly showed that cationization could greatly promote the production of ROS. The cationization of DTPAPy also further promoted the formation of ^1^O_2_ and free radicals (Figure 16c). Then the density functional theory (DFT) method was used for theoretical calculation. As shown in Figure 16d,e, the HOMO and LUMO of DTPAPy were distributed on TPA and dicyanisophosphonone, and the separation degree was poor. Cationization changes the pyridine segment from an electron donor to an electron acceptor. Therefore, the LUMO of DTPAPyPF_6_ was concentrated in the pyridine cation portion, resulting in a distinct separation of HOMO‐LUMO distribution. The ΔE_S‐T_ bandgap of DTPAPyPF_6_ (0.4399 eV) was much smaller than that of DTPAPy (0.9530 eV), so DTPAPyPF_6_ should have better ISC capability than DPTAPy.

**Figure 17:**
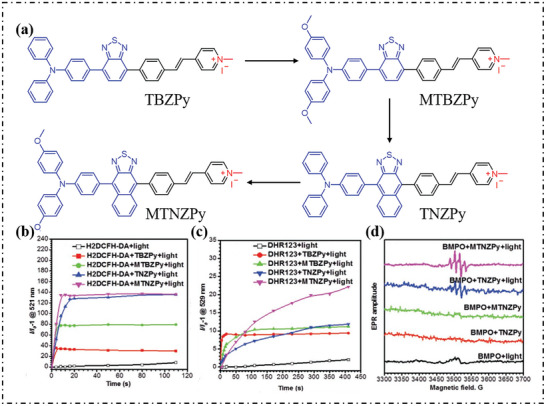
a) Chemical structures of TBZPy, MTBZPy, TNZPy, and MTNZPy. b) ROS generation by the four PSs (10 µm) upon white light irradiation using H_2_DCF‐DA (5 µm) as an indicator. c) plot of the relative emission intensity (*I/I_0_*) of a DHR123 (10 µm) solution containing PSs (10 µm) versus the irradiation time. d) EPR signals of BMPO for free radical ROS characterization. Reproduced with permission.^[^ ^76^ ^]^ Copyright 2020, Wiley‐VCH GmbH.

Tang's group prepared four anion‐π^+^ AIEgens (**Figure** **17**a).^[^ ^76^ ^]^ The enhancement of the electron‐donating ability of anion‐π^+^ AIEgens follows the order of TBZPy < MTBZPy < TNZPy < MTNZPy, where the iodide anion and the strongly reducing co‐donor in these four molecules create electron‐rich conditions in the aggregate microenvironment and provide electrons for excited PSs. Through the detection of ROS of four molecules, it can be seen that the trend of total ROS production was TBZPy < MTBZPy < TNZPy < MTNZPy (Figure 17b). The DHR123 fluorescence intensity of TBZPy and MTBZPy increased sharply and then leveled off, however, the intensity of TNZPy and MTNZPy increased steadily and eventually exceeded TBZPy and MTBZPy (Figure 17c). Further EPR experiments also verified the generation of O_2_·^−^ (Figure 17d).

### 3.3 Introduce an Enhanced Electron Transfer Capacity Primitive

The introduction of appropriate substrates with suitable oxidation‐reduction potential is an effective strategy to facilitate the electron transfer process, thereby promoting the generation of type‐I ROS. Among them, quinone and its derivatives, with redox cycle and electron transfer capacity, are the most promising candidates.^[^ ^77^, ^78^, ^79^ ^]^

**Figure 18:**
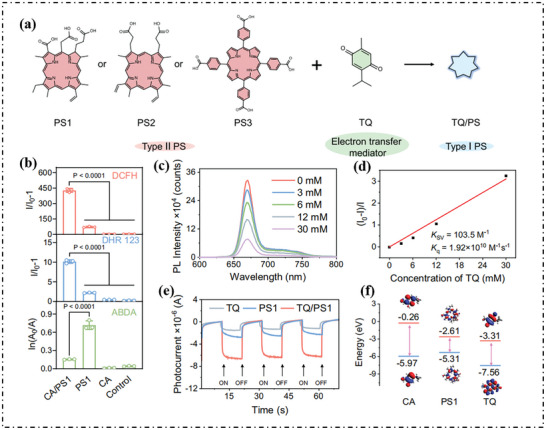
a) Chemical structures of type‐II PSs and TQ. b) Summary of different types of ROS generation in the presence of ROS detection probes alone and probes with different treatments. Data in b is presented as mean ± SD derived from *n* = 3 independent samples. c) The emission spectra change of PS1 (10 µm) with increasing concentrations of TQ (0‐30 mm) under excitation at 405 nm in DMF. d) Stern‐Volmer plot of fluorescence intensity change of PS1 against TQ in DMF. e) Photocurrent responses of PS1, TQ, and TQ/PS1 systems. f) HOMO and LUMO energy levels of CA, PS1, and TQ.^[^ ^80^ ^]^ Copyright 2024, Nature Publishing Group. Open Access.

Liu's group combined the natural substrate carvacrol (CA) with classic type‐II PSs (PS1, PS2, PS3).^[^ ^80^ ^]^ CA served as a biologically active molecule sensitive to ^1^O_2_ and could be activated in situ. Subsequently, it was converted into tetrahydrobiopterin (TQ). TQ acted as an electron transfer mediator that facilitated the electron transfer between PSs and O_2_, leading to enhanced type‐I and efficient generation of O_2_·^−^ (**Figure** **18**a). As shown in Figure 18b, when CA/PS1 was present, the solution of DCFH showed a fluorescence enhancement of ≈433‐folds, DHR123 of 5.5‐folds compared with PS1, suggesting that the combination of PS1 and CA can bring about an unexpected type‐I ROS generation capacity for type‐II PS1. With the increase of TQ concentration, the fluorescence of PS1 was quenched (Figure 18c). The quenching constant (*K*_q_) calculated by the Stern‐Volmer equation was 1.92 × 10^10^ M^−1^ S^−1^ (Figure 18d), further proved that TQ could effectively quench the excited state of PS1 through the ET process. Meanwhile, the photocurrent response of PS1, TQ, and TQ/PS1 systems was studied. As shown in Figure 18e, the TQ/PS1 system exhibited a stronger photocurrent than either PS1 and TQ alone, indicating an enhanced charge transfer rate. In addition, the LUMO level of PS1 was −2.61 eV, between TQ (−3.31 eV) and CA (−0.26 eV) (Figure 18f), which was consistent with the spectral result.

**Figure 19:**
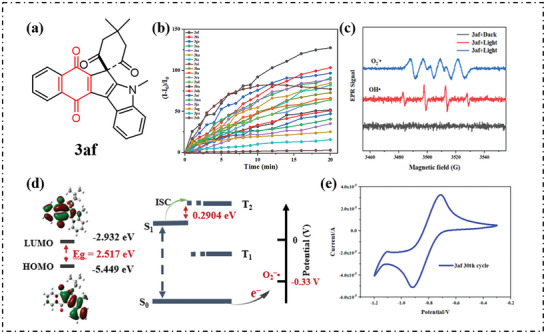
a) Chemical structure of 3af. b) Fluorescence intensity net change *(I – I_0_)/I_0_* at 521 nm for the DCFH indicator with compound 3 upon 660 nm irradiation (500 mW cm^−2^). c) EPR spectra of DMPO/O_2_·^−^ and DMPO/OH· for 3af under 650 nm irradiation. d) HOMO–LUMO distribution at S_1_, and the energy gap between S_1_ and T_2_ for 3af from TD‐DFT. e) 30th cyclic voltammogram of 3af in DCM. Reproduced with permission.^[^ ^81^ ^]^ Copyright 2024, The Authors. Royal Society of Chemistry.

Our group designed and synthesized a series of new quinone‐fused cyclopenta[2,1‐b] indoles as pure type‐I PSs (structure of 3af shown in **Figure** **19**a).^[^ ^81^ ^]^ As shown in Figure 19b, 20 compounds were tested, and all compounds produced large amounts of ROS. The type of ROS was further verified using EPR spectroscopy, and resonant signals were observed with strengths of 1:1:1:1 and 1:2:2:1, which are consistent with O_2_·^−^ and OH· signals (Figure 19c). As shown in Figure 19d, the HOMO of 3af was mainly located in the indole unit, and the LUMO was distributed in the naphthoquinone part. The energy levels of singlet and triplet states were further calculated, and ΔE_S1‐T2_ of 3af was 0.2904 eV, which facilitates the ISC process. In order to evaluate whether 3af can retain the good electron transfer ability of quinone, electrochemical experiments were carried out by CV. As shown in Figure 19e, 3af exhibited electrochemical properties similar to that of p‐benzoquinone, with an obvious reduction peak at −0.935 V. Given the reduction potential of O_2_ (*E_red_* (O_2_/O_2_·^−^) = −0.33 V), the reduction potential in the ground state of the 3af *E_red_* (PS/PS·^−^) was significantly lower than −0.33 V, showing excellent electron transfer ability.

**Figure 20:**
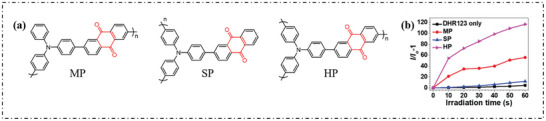
a) Chemical structure of MP, SP, and HP. b) Relative changes in the PL intensity of DHR123 in the presence of MP, SP, and HP under 530 nm laser irradiation (100 mW cm^−2^). Reproduced with permission.^[^ ^82^ ^]^ Copyright 2022, Royal Society of Chemistry.

Using trianiline (TPA) as electron donor and anthraquinone (AQ) as acceptor, Li's group synthesized three polymeric PSs with different main chains, namely main chain polymer (MP), side chain polymer (SP) and overspent chain polymer (HP). Their structures are shown in **Figure** **20**a.^[^ ^82^ ^]^ As shown in Figure 20b, MP, SP, and HP can increase the PL intensity of DHR123 by 56, 12, and 116 times, respectively, indicating that these three PSs can produce O_2_·^−^.

**Figure 21:**
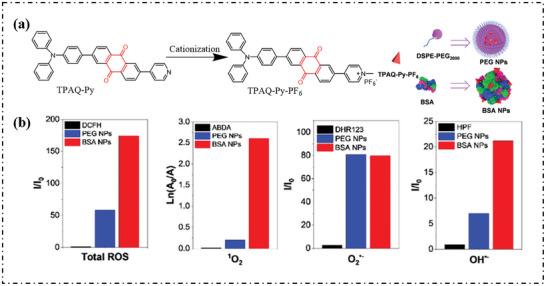
a) Cationized molecular engineering strategy and schematic diagram of preparation of BSA NPs and PEG NPs based on TPAQ‐Py‐PF_6_. b) The ability of PEG NPs and BSA NPs to generate different ROS under 2 min white light irradiation. Reproduced with permission.^[^ ^69^ ^]^ Copyright 2023, American Chemical Society.

Tang's group combined the three categories in the direct strategy and designed a cationized (providing an electron‐deficient intermediate transmitter) quinone derivative (introducing an enhanced electron transfer), and self‐assembled with BSA (forming an electron‐rich microenvironment) to obtain a PSs system with a large proportion of type‐I ROS (**Figure** **21**a).^[^ ^79^ ^]^ Similar to the previous example, it was found that cationization could increase the production of ROS. Meanwhile, compared with traditional polymer coating methods, the immobilization and electric‐rich of BSA can promote ROS generation to a large extent, especially OH·, as shown in Figure 21b.

The direct strategy improves the efficiency of electron transfer by forming an electron‐rich microenvironment, providing an electron‐deficient intermediate transmitter, and introducing an enhanced electron transfer capacity primitive. However, these approaches face challenges in balancing effective electron transfer with the oxidation of biomolecules. In particular, although a strong electron donor can form an electron‐rich microenvironment that promotes the effective electron transfer of PSs, it also lowers the oxidation potential of PSs, resulting in weaker photooxidation ability of PSs to biomolecules in type‐I PDT. Additionally, the introduction of electron‐deficient intermediate transmitter may enhance the reactivity of PSs, leading to undesirable reactions with non‐target biomolecules. This can cause side effects or reduce the accuracy of treatment. Therefore, optimizing PS performance requires more refined molecular design and modification to minimize the side effects while improving the therapeutic efficacy, which will facilitate the further development and improvement of photodynamic therapy technologies.

## 4 Summary and Outlook

Over the past few decades, the PDT approach has achieved significant progress and development. While numerous PDT drugs have been approved for clinical trials or applications, they are not yet the preferred treatment option currently. The high oxygen dependence of type‐II PSs severely limits their applications in tumor therapy, so the hypoxia‐resistant type‐I PSs are widely concerned. The methods of constructing type‐I photosensitizer are summarized in this review from two perspectives. Frist, indirect strategy reduces the energy of T_1_ state to be lower than the energy required to produce ^1^O_2_ by EET process. Second, direct strategy increases the electron transfer efficiency of ET process, including i) forming an electron‐rich microenvironment using BSA, BP5A, heteroatoms, and heterocyclic rings; ii) providing an electron‐deficient intermediate transmitter using electron acceptors and molecular cationization; iii) introducing an enhanced electron transfer capacity primitive with quinone and its derivatives.

Despite these advances, there are still some challenges in the type‐I PSs. The biggest obstacle is the ambiguous design strategy. Although in this review, we have detailed classification, listing, and elaboration of the current design concept of type‐I PSs, which can provide guidance in this field, there are still the following problems that waiting to be solved.

In indirect strategy, achieving efficient OH· generation requires both low T_1_ and high ISC efficiency. Precise regulation of T_n_ energy levels should be realized without compromising other photophysical properties, which rely heavily on molecular designing. In contrast, direct strategy has a clear understanding about the method and mechanism of constructing electron‐rich environment and electron‐deficient structure. However, type‐I and type‐II ROS often coexist, making it challenging to achieve pure type‐I PDT. Further efforts should be focused on enriching the material system. However, electron transfer capacity primitive is still very rare, with only quinone structures explored so far. New intermediates with strong electron transfer ability need to be developed and designed for rational application in type‐I PSs, which is the most effective way to build type‐I PSs.

In addition to the design strategy of type‐I PSs, which were mainly discussed in this review, the performance and application of PSs are also important. For example, the absorption /fluorescence of type‐I PSs should be further extended to the NIR‐II window (1000–1700 nm), which can enhance tissue penetration. PSs that can be synergistic with other therapeutic methods (such as chemotherapy, immunotherapy, etc.) should also be designed to improve the therapeutic effect. Additionally, more methods to determine OH· and O_2_·^−^ should be developed. The most commonly used methods reported in the literature are ESR and fluorescent probes to qualitatively determine the production of OH· and O_2_·^−^, which means that our understanding of type‐I process and mechanism has certain limitations, and more determination methods need to be further studied and developed.

This field holds both challenges and opportunities, especially in clinical trials. This review summarizes the recent strategies of type‐I PSs from two aspects, indirect and direct strategy, which may help to promote the rapid development of type‐I PSs with clearer design principles and broader molecular platforms. It can be anticipated that above challenges will be addressed in the future with the rapid development of type‐I PSs in PDT.

## Conflict of Interest

The authors declare no conflict of interest.
